# Mutagenesis Study Reveals the Rim of Catalytic Entry Site of HDAC4 and -5 as the Major Binding Surface of SMRT Corepressor

**DOI:** 10.1371/journal.pone.0132680

**Published:** 2015-07-10

**Authors:** Gwang Sik Kim, Ha-Eun Jung, Jeong-Sun Kim, Young Chul Lee

**Affiliations:** 1 Hormone Research Center, School of Biological Sciences and Technology, Chonnam National University, Gwangju, 500–757, Republic of Korea; 2 Department of Chemistry and Institute of Basic Sciences, Chonnam National University, Gwangju, 500–757, Republic of Korea; Centro de Biología Molecular Severo Ochoa (CSIC-UAM), SPAIN

## Abstract

Histone deacetylases (HDACs) play a pivotal role in eukaryotic gene expression by modulating the levels of acetylation of chromatin and related transcription factors. In contrast to class I HDACs (HDAC1, -2, -3 and -8), the class IIa HDACs (HDAC4, -5, -7 and -9) harbor cryptic deacetylases activity and recruit the SMRT-HDAC3 complex to repress target genes *in vivo*. In this regard, the specific interaction between the HDAC domain of class IIa HDACs and the C-terminal region of SMRT repression domain 3 (SRD3c) is known to be critical, but the molecular basis of this interaction has not yet been addressed. Here, we used an extensive mutant screening system, named the “partitioned one- plus two-hybrid system”, to isolate SRD3c interaction-defective (SRID) mutants over the entire catalytic domains of HDAC4 (HDAC4c) and -5. The surface presentation of the SRID mutations on the HDAC4c structure revealed that most of the mutations were mapped to the rim surface of the catalytic entry site, strongly suggesting this mutational hot-spot region as the major binding surface of SRD3c. Notably, among the HDAC4c surface residues required for SRD3c binding, some residues (C667, C669, C751, D759, T760 and F871) are present only in class IIa HDACs, providing the molecular basis for the specific interactions between SRD3c and class IIa enzymes. To investigate the functional consequence of SRID mutation, the *in vitro* HDAC activities of HDAC4 mutants immuno-purified from HEK293 cells were measured. The levels of HDAC activity of the HDAC4c mutants were substantially decreased compared to wild-type. Consistent with this, SRID mutations of HDAC4c prevented the association of HDAC4c with the SMRT-HDAC3 complex *in vivo*. Our findings may provide structural insight into the binding interface of HDAC4 and -5 with SRD3c, as a novel target to design modulators specific to these enzymes.

## Introduction

Chromatin is the basic structure of the eukaryotic chromosomes, formed as an array of nucleosomes composed of histone octamers wrapped with 146 bp of DNA [1]. The tight binding of DNA with histones in condensed chromatin acts as an obstacle for DNA binding of various proteins involved in DNA metabolism, including transcription. In this regard, chromatin remodeling and histone tail modifications are major mechanisms to convert chromatin status from closed conformation (inactive) to open conformation (active), or vice versa [2]. Epigenetic control of gene expression is achieved via the complicated interplay among DNA methylation, histone tail modifications, and chromatin remodeling. The consequential chromatin status is thought to play a major role in X-inactivation, heterochromatin formation and maintenance, and homeotic gene expression during early development in animals [3]. Currently, epigenetic control is also regarded as a general mechanism for the gene-specific regulation of eukaryotic transcription, as most epigenetic modifications were proven to be reversible and to arise in a promoter-specific manner [4,5]. Among these dynamic epigenetic markers, acetylation and deacetylation occur at specific lysine residues within the N-terminal tail of nucleosomal histones through the opposite actions of two respective families of enzymes, the histone acetyltransferases (HATs) and histone deacetylases (HDACs) [2]. In general, histone acetylation by HATs acts as an active mark for gene transcription, whereas histone deacetylation by HDACs correlates with transcriptional repression. Therefore, the transcriptional activity of a specific gene, or the compaction level of a local chromatic region is established through the balanced actions of HAT and HDAC enzymes [6]. HDACs play a central role in the regulation of many biological processes, such as the cell-cycle, cell differentiation and survival [6,7]. Genetic mouse models revealed HDACs to be essential in embryonic development, cardiovascular health and energy metabolism [8,9]. In this respect, the selective blocking of specific HDAC function has a great therapeutic impact on many diseases, including cancer, cardiovascular, neurodegenerative, and metabolic disorders [10–13]. At present, a variety of HDAC inhibitors are under clinical investigation, while two HDAC inhibitors, SAHA (vorinostat) and FK228 (romidepsin), were already approved for the treatment of cutaneous T-cell lymphomas [14].

To date, 18 kinds of mammalian HDACs have been identified and classified into four classes based on their sequence similarities and domain structure [15]. The class I HDACs (HDAC1, -2, -3, and -8) are mammalian homologues of the yeast Rpd3 corepressor, and contain only the HDAC domain with a size of about 350 amino acids [16]. They are nuclear proteins with ubiquitous expression in most cell types. The class II HDACs (HDAC4, -5, -6, -7, -9, and -10) share homology with yeast Hda1p, and display cell type-specific expression [17]. For example, HDAC4, -5 and -9 are known to be enriched in the heart, skeletal muscle and brain tissues. Class II enzymes can be further subdivided into class IIa (HDAC4, -5, -7, and -9) and IIb (HDAC6 and -10) according to their modular structure. In addition to the C-terminal HDAC domain, class IIa HDACs have a long-extending N-terminal adaptor domain, which is targeted by various transcription factors and regulatory signals [17]. Class IIa enzymes can shuttle between the cytoplasm and nucleus based on the presence or absence of modifications to specific residues in the adaptor domain, induced by external stimuli. Class IIb enzymes (HDAC6 and -10) have a characteristic long extension of the C-terminal tail domain, and are typically found in the cytoplasm. In particular, HDAC6 is a microtubule-associated deacetylase with dual HDAC domains, compared with the single domain of HDAC10 [18]. Class IV includes only HDAC11, which shows strong sequence similarity to the class I HDACs and is predominantly located in the nucleus [19]. The class I, II and IV HDACs are zinc-dependent enzymes, harboring a common enzymatic mechanism focused on zinc-catalyzed hydrolysis of the acetyl-lysine amide bond [20]. In contrast, the class III HDACs (sirtuin family) require NAD^+^ as a cofactor for catalytic function, and are not sensitive to the HDAC inhibitors effective on class I and II enzymes [8].

HDACs are unable to bind to DNA by themselves. They exist as components in a variety of multiprotein complexes that are recruited to target promoters via interactions with many DNA-binding factors, such as unliganded nuclear receptors, the E-box binding factors, and the methylcytosine binding protein [21,22]. For example, HDAC1 and HDAC2 are found in three distinct corepressor complexes, called SIN3A, NURD/Mi2 and CoREST [23,24]. NCoR (nuclear receptor-corepressor) and SMRT (silencing mediator for retinoid and thyroid receptor) are ubiquitously expressed corepressor proteins containing three autonomous repression domains (RD1 to RD3) in their N-terminal regions [25,26]. Each of the repression domains plays a non-redundant role in the platform for recruitment of various DNA-binding repressors or corepressors, including HDACs [27]. HDAC3 is exclusively found in SMRT/NCoR complexes, with which association is achieved through the conserved deacetylase activating domain of NCoR/SMRT [28,29]. The activation of HDAC3 through this association was proven to be essential for transcriptional repression by certain nuclear receptors, including thyroid hormone receptor and Rev-erbs [30,31]. A recent study from the Lazar group indicated that a deacetylase-independent but NCoR-dependent function of HDAC3 is essential for the transcriptional regulation of hepatic genes, highlighting the non-enzymatic roles of HDAC3 in liver metabolism [32]. As mentioned previously, class IIa HDACs have a modular structure composed of N-terminal adaptor and C-terminal HDAC domains. The adaptor domain recruits many corepressors including BCoR, CtBP and HP1 as effector molecules, as well as various repressors such as MEF2, FOXP3 and RUNX2 as target transcription factors [17]. Consistent with this, knockout mice models demonstrated that the loss of class IIa HDACs can lead to abnormalities during skeletogenesis and heart development [11,33–35]. Notably, the C-terminal region of SMRT RD3 (SRD3c) specifically interacts with the HDAC domain of class IIa HDACs, but not with class I enzymes [27]. This observation strongly suggested that class IIa HDACs function as a bridge between target repressor proteins and the SMRT/NCoR-HDAC3 complex via independent interactions occurring through the two domains [36].

Structural and functional analyses of inhibitor or substrate-bound HDACs have been attempted. The crystal structure of HDAC8 bound with acetylated peptide substrate revealed that the residues (His142, His143, Asp176, Asp183 and Tyr306) around the catalytic cavity interact with one water molecule and zinc ion [37]. These interactions are conserved among the catalytic zinc-binding pockets of class I enzymes, and are important for substrate recognition and zinc-catalyzed hydrolysis of the acetyl-lysine amide bond of the peptide substrate [37,38]. The crystal structures of the catalytic domain of HDAC7, as well as inhibitor bound forms of HDAC4 and -7 were also investigated [39,40]. These studies revealed that HDAC4 and -7 harbor a second zinc-binding domain, adjacent to the zinc-containing catalytic domain. This class IIa HDAC-specific region is well conserved in other class IIa HDACs, and has been implicated to have regulatory and structural roles [39–41]. Another structural feature of class IIa HDACs is the unique topology of the active site in an enlarged active site pocket [39]. In the class IIa enzymes, the catalytic active site contains a histidine residue (His976 of HDAC4, His1006 of HDAC5 and His843 of HDAC7) instead of the tyrosine residue conserved in class I enzymes (Tyr298 of HDAC3 and Tyr306 of HDAC8), which functions as a transition-state stabilizer of the catalytic reaction. Interestingly, a His-976-Tyr mutation in HDAC4 dramatically increases its enzymatic activity to the level of class I enzymes [42], explaining the reason why class IIa HDACs purified from bacteria harbor very low deacetylase activity toward acetylated lysine as compared with class I enzymes. Recently, proteomic analysis revealed that the absence of HDAC4 had no effect on the acetylation profile of the murine neonate brain, providing *in vivo* evidence that HDAC4 may not function as a lysine deacetylase in this tissue [43]. Considering the cryptic activity of class IIa enzymes, the HDAC activity shown by the endogenous class IIa HDAC complex purified from mammalian cells is supplied by the SMRT/NCoR-HDAC3 complex via its association with the HDAC domain [36]. In this regard, the catalytic domain of class IIa HDACs is a pseudo-HDAC domain that functions as a SMRT-recruiting module, independent of the deacetylase activity.

The structural differences between class I and IIa enzymes strongly suggest that the structural zinc-binding domain, which is specific to class IIa HDACs, may participate in their specific interactions with the SRD3c region. To test this possibility, we employed a ‘one- plus two-hybrid system’ (OPTHiS) to obtain SRD3c interaction-defective (SRID) mutants over the entire catalytic domains of HDAC4 (HDAC4c) and HDAC5 (HDAC5c). Surprisingly, the surface presentation of the SRID mutations on the HDAC4c structure revealed that most of the mutations were mapped to the rim surface of the catalytic entry site, rather than the structural zinc-binding domain, as the mutational hot-spot. Furthermore, some mutant residues (C667, C669, C751, D759, T760 and F871) were found to be present only in class IIa HDACs, providing the molecular basis by which SRD3c specifically interacts with class IIa HDACs, but not with class I enzymes.

## Materials and Methods

### Plasmids

**Fig 1:**
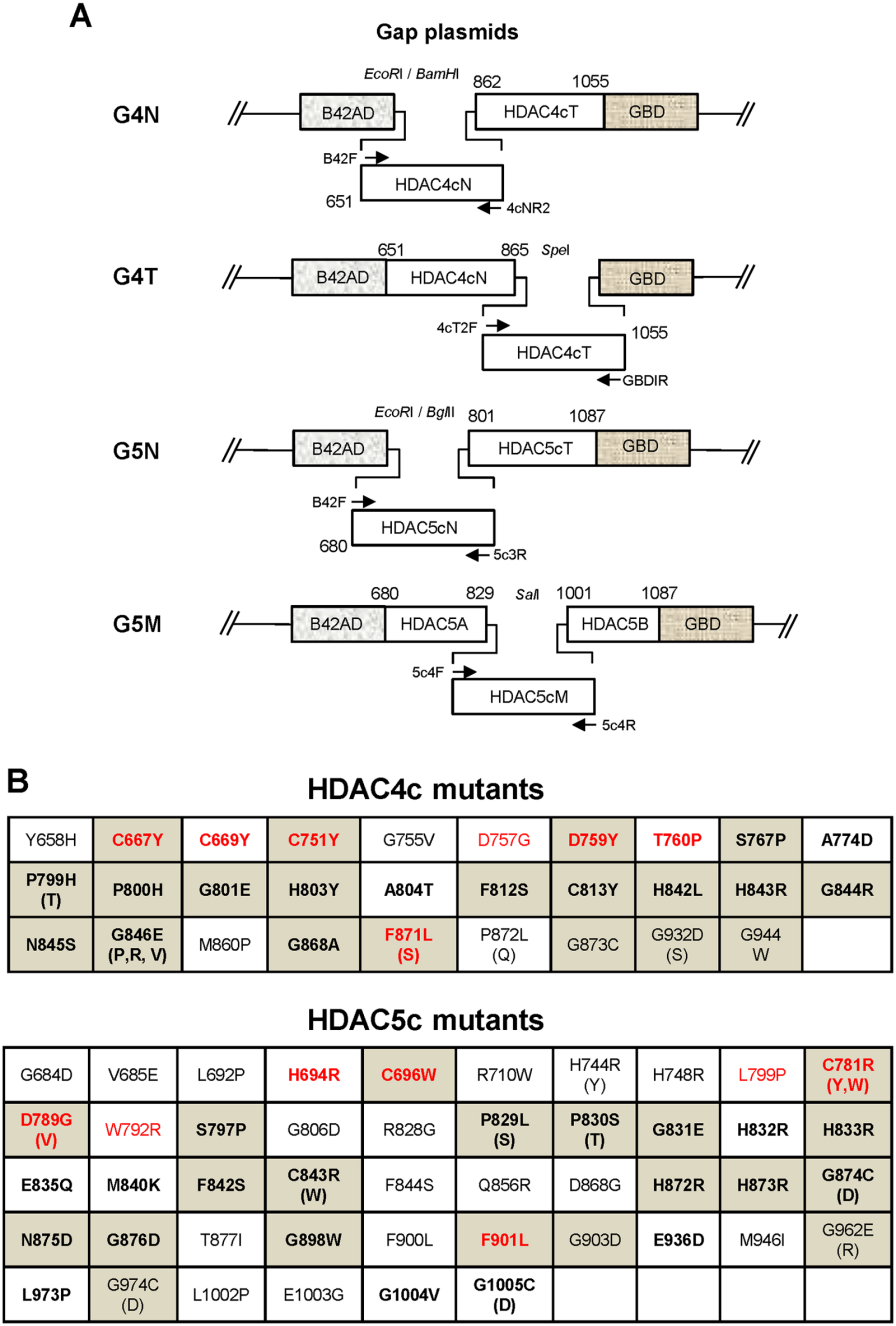
SRID mutants of HDAC4c and -5c obtained by Partitioned OPTHiS. **(A)** Schematic depiction of gap plasmids and targeted regions of HDAC4c and -5c for the SRID mutant screening by Partitioned OPTHiS. The gap plasmids G4N and G4T were used for the screening of SRID mutants targeted for the HDAC4cN (amino acids 651 to 865) and HDAC4cT (amino acids 865 to 1055) regions of HDAC4c, respectively. For HDAC5c, the gap plasmids G5N and G5M were used to isolate SRID alleles present in the HDAC5cN (amino acids 680 to 831) and HDAC5cM (amino acids 829 to 1010) regions of HDAC5c, respectively. The linearized gap plasmids and mutagenic PCR products were prepared with the use of the indicated restriction enzymes (top) and the denoted primer sets (arrow), respectively. **(B)** The list of SRID alleles of HDAC4c (upper) or HDAC5c (lower). The residues in gray boxes were commonly found between SRID mutants of HDAC4c and -5c based on sequence alignment. The residues existing on the surface of HDAC4c are presented in bold, and the class IIa HDAC-specific residues are shown in red.

To construct the bait plasmid pRS325LexA-RD3c used in OPTHiS, the human SRD3c region (amino acids 1281 to 1504) was amplified by PCR and inserted into the BglII/NcoI sites of the pRS325LexA vector. The catalytic domains of human HDAC4 (amino acids 650 to 1055) and -5 (amino acids 680 to 1087) were obtained from pBJ5.1-Flag clones by PCR and cloned into the EcoRI/BamHI sites of pRS324UBG to make pRS324UBG-HDAC4c and -5c, respectively. To isolate SRID alleles of HDAC4c and HDAC5c, G4N, G4T, G5N, and G5M plasmids were constructed and used as gapped plasmids for OPTHiS screening (Fig 1A). The G4N and G4T plasmids were made by inserting PCR fragments of HDAC4cT (amino acids 862 to 1055) and HDAC4cN (amino acids 651 to 862) into the EcoRI/BamHI sites of pRS324UBG, respectively. In the case of the G5N plasmid, the PCR fragment of HDAC5cT (amino acids 801 to 1087) was digested by EcoRI/BamHI and inserted into the corresponding sites of pRS324UBG. To construct the G5M plasmid, the HDAC5A (amino acids 680 to 829) and -5B (amino acids 1001 to 1087) fragments of HDAC5c were obtained by PCR amplification and cloned into the EcoRI/BamHI sites of pRS324UBG by three-piece ligation. pcDNA3-HA-HDAC4c and -HDAC5c were made by inserting the respective PCR fragments of HDAC4c and HDAC5c into the EcoRI/XbaI sites of the pcDNA3-HA vector. For bimolecular fluorescence complementation (BiFC) assay, KGN-MC-SRD3c (amino acid 1281 to 1504) was constructed by subcloning the KpnI/XhoI fragment from pcDNA3-HA-SRD3c into the KGC-MC vector (MLB International Corporation). As the first step to making KGC-MC-HDAC4c, pBS-HDAC4c was prepared by inserting the EcoRI/XbaI fragment of pcDNA3-HA-HDAC4c into the pBlueScript vector (Stratagene). The KpnI/NotI fragment of pBS-HDAC4c and the KpnI fragment of pcDNA3-HA-HDAC4c were then sequentially were inserted into the KpnI/NotI sites of KGC-MC (MLB international corporation), resulting in KGC-MC-HDAC4c. To construct KGC-MC-HDAC5c, the KpnI/XhoI fragment was obtained from pcDNA3-HA-HDAC5c and subcloned into the corresponding sites of KGC-MC. For GST-pull down assay, pGEX4T-SRD3c was constructed by subcloning the EcoRI/XhoI fragment from pcDNA3-HA-SRD3c into pGEX4T-1 (Amersham Biosciences). The SRID mutants of HDAC4c and -5c isolated by OPTHiS were subcloned into pcDNA3-HA (for *in vitro* translation) or KGC-MC (for BiFC assay) using the appropriate enzyme sites from the pRS324UBG version, respectively. All constructs were confirmed by DNA sequencing.

### Mutagenic PCR and Partitioned OPTHiS Screening

Random mutagenesis of HDAC fragments and OPTHiS screening were conducted as previously described in Kim et al. [44,45]. Briefly, mutagenic PCR fragment containing the HDAC4cN or HDAC4cT regions were amplified in the presence of 0.1 mM MnCl_2_ using pRS324UBG-HDAC4c as templates with oligomer pairs B42F/4cNR2 for HDAC4cN and 4cT2F/GBDIR for HDAC4cT, respectively. In the case of random mutagenesis of the HDAC5cN and HDAC5cM fragments, PCR was conducted in the presence of 0.05 mM MnCl_2_ using pRS324UBG-HDAC5c as templates with oligomer pairs B42F/5c3R for HDAC5cN and 5c4F/5c4R for HDAC5cM, respectively. For OPTHiS screening to obtain SRID mutants, the yeast cell libraries containing HDAC mutants were constructed by a single step method via *in vivo* gap repair [45]. The four kinds of mutagenic PCR products (1 μg) were co-transformed with the corresponding linearized gap plasmids (250 ng) into yeast strain YOK400 (*MATa*, *leu2*, *trp3*, *ura3*, *lexA*_*op*_ *-LEU2*, *UAS*_*GAL*_ *-HIS3*) carrying the pSH18-34 reporter as well as the bait plasmid pRS325LexA-SRD3c. The transformants were grown for 3 days at 30°C in synthetic glucose medium lacking histidine for the positive selection of intact HDAC fusions using the endogenous *UAS*_*GAL*_-*HIS3* reporter gene [44]. Among the surviving transformants, SRID mutants were selected by isolating white colonies on X-gal plates using the episomal two-hybrid reporter (*lexA*_*op*_ *-LacZ*). For the actual screening experiment, a large number of transformants was obtained from several batches of standard-scale transformation. Subsequent verification of the HDAC mutants defective in SRD3c binding was carried out as described previously [45].

### Cell Culture and Transient Transfection Assay

HEK293 cells were maintained in DMEM (Welgene) supplemented with 10% fetal bovine serum (Welgene) and antibiotics-antimycotic (Gibco). Cells were seeded in 24-well plates with 4–8 X 10^4^ cells/well on the day prior to transfection. Transient transfections were performed using the SuperFect (QIAGEN) or TurboFect (Fermentas) systems, as described in the manufacturer’s instructions. After 24 h of transfection, cell lysates were prepared with RIPA buffer [50 mM Tris-HCl (pH 8.0), 5 mM EDTA, 150 mM NaCl, 1% NP-40, 1 mM PMSF] and used for luciferase and β-galactosidase assays. Luciferase activity was normalized to β-galactosidase activity for each sample.

### BiFC Assay

To examine the protein interactions of SRD3c with HDAC4c or HDAC5c mutants in living cells, bimolecular fluorescence complementation (BiFC) assays were carried out using a Fluo-Chase kit (Amalgaam), according to the manufacturer’s manual. Briefly, SRD3c and HDAC proteins were fused to the N- or C-terminal portions of Kusabira Green protein, resulting in KGN-SRD3c and KGC-HDAC4c or -5c constructs, respectively. The KGN-SRD3c was coexpressed with KGC-HDAC4c or -5c in HEK293 cells using the SuperFect system in a 96-well black plate (SPL life science). After 48 h of transfection, the fluorescent signals (excitation wavelength: 494 nm, emission wavelength: 538 nm) from the cell lysates were measured using a fluorescence spectrophotometer (Molecular Devices, Spectra max GEMINIXPS). The quantitation experiments were repeated two times for the triplicated samples.

### Confocal Laser Scanning Microscopy

HEK293 cells were grown on 8-well slide plates (SPL Life science) and cotransfected with KGN-SRD3c and KGC-HDAC mutants using the SuperFect system. After 48 h of transfection, cells were incubated in 4% paraformaldehyde for 10 m at room temperature for fixation. The cells were then washed with 1X PBS, mounted onto micro cover-slides, and observed for green fluorescence using a laser-scanning confocal microscope (Leica TCS SPE).

### Immunoprecipitation, *In Vitro* HDAC Assay and Immunoblot Analysis

Human HDAC3 was coexpressed with HA-tagged versions of the wild-type or HDAC4c mutants in HEK293 cells using the SuperFect system. After 48 h of transfection, the whole cell lysates (400–600 μg) were prepared with RIPA buffer and mixed with 30 μl (50% slurry) of agarose beads coupled with anti-HA-monoclonal antibody (eBioscience). After overnight incubation at 4°C, the beads were washed three times with RIPA buffer. For *in vitro* HDAC assay, immuoprecipitates (beads) were mixed with fluorescent-coupled Lys acetamide substrate (BioVision. Cat. No. K330-100) and transferred into a 96- well black plate. HDAC activity assay was carried out according to the manufacturer’s instructions, and the fluorescence signal (excitation wavelength: 380 nm, emission wavelength: 460 nm) was measured using a fluorescence plate reader. For co-immunoprecipitation assay, the bound proteins were eluted from the immunoprecipitates using 0.1M glycine-acetate (pH 3.0), after which the eluents were precipitated using tricholoroacetic acid. The precipitated proteins were resolved on SDS-PAGE gels and analyzed for the presence of HA-HDAC4c and HDAC3 by immunoblot using anti-HA mouse (Cell Signaling, #2367; 1:3,000 dilution) and anti-HDAC3 rabbit (Abcam, Ab16047; 1:1,000 dilution) antibodies, respectively. Immunoblots were developed using the Optiblot ECL ultra detection kit (Abcam, Ab133409), and images were captured using an HP imaging system.

### Yeast Two-Hybrid and GST Pull-Down Assay

Yeast strain EGY48 containing pSH18-34 (8X *LexA*_op_-*LacZ* reporter plasmid) was co-transformed with the expression plasmids for LexA-SRD3c (pEG202-SRD3c as bait) and for wild-type or mutants of HDACs fused between B42AD and GBD (pRS324UBG-HDAC4c or -5c as a prey) by the lithium acetate method. Liquid assays for β-galactosidase activity were conducted at least three times, as described previously [44]. Detailed information for the expression and purification of GST alone and GST-fused proteins were described in our previous report [45]. The radiolabeled HDAC proteins were added to similar amounts of GST or GST-SRD3c proteins (2–3 μg) bound to glutathione-agarose beads pre-equilibrated with buffer A [150 mM Tris-HCl (pH 7.9), 5% glycerol, 1 mM EDTA, 1 mM dithiothreitol, 1x protease inhibitor, 0.01% NP-40, 150 mM KCl] in a final volume of 250 μl. The beads were washed three times in the same buffer and the bound radiolabeled proteins were analyzed by SDS-PAGE followed by autoradiography.

### Statistical Analysis

All quantitation experiments were repeated two or three times for the triplicated samples. The student’s *t*-test was used to measure statistically significant differences between wild-type and mutant groups in the corresponding graphs and their *p*-values were summarized in S3 Table.

## Results

### Partitioned OPTHiS for the Isolation of SRID Alleles over the Entire Catalytic Domain of HDAC4 and -5

As mentioned, the SRD3c region specifically interacts with class IIa HDACs, but not with class I enzymes, strongly suggesting that a class IIa HDAC-specific region, such as the structural zinc-binding domain, may be involved in this interaction. To gain an understanding of the molecular basis of the specific interactions between SRD3c and class IIa HDACs, we employed the one- plus two-hybrid system (OPTHiS) to map the interaction surface of class IIa HDACs with SRD3c [44]. OPTHiS is a novel yeast genetic system designed to efficiently select for missense mutant alleles which specifically disrupt a known protein-protein interaction. To operate OPTHiS, we first tried to define the minimal region of the catalytic domain of HDAC4 (HDAC4c) essential for SRD3c binding. To accomplish this, serial truncation mutants of HDAC4c were made and tested for SRD3c binding in the yeast two-hybrid system. We found that the entire region of HDAC4c (amino acids 651 to 1055) was necessary and sufficient for optimal interaction with SRD3c (data not shown), indicating that the intactness of the SRD3c-binding surface of HDAC4 requires the three-dimensional structure, rather than a short motif of HDAC4c. We considered that the whole region of HDAC4c (about 400 amino acids) is too long to maintain the optimal mutation rate by OPTHiS [44]. Therefore, the HDAC4c domain was further divided into two regions (HDAC4cN, HDAC4cT) with a size of about 200 amino acids, and each region was independently screened for SRID mutants by OPTHiS. As the first step to isolate the full-length alleles of missense mutants, each mutagenic PCR fragment corresponding to the HDAC4cN or HDAC4cT region was co-transformed into yeast strain YOK400 with the linearized gap plasmids G4N and G4T, respectively. In this case, the gap plasmids G4N and G4T were designed to contain HDAC4cT or HDAC4cN fragments, respectively, between the B42AD and GBD regions of the pRS324UBG plasmid (Fig 1A). After co-transformation into yeast, *in vivo* gap repair by the homologous recombination between PCR fragment and the linearized gap plasmid produced a full-length HDAC4c domain, inserted between B42AD and GBD, which harbored missense mutation(s) in the targeted region. We named this strategy “Partitioned OPTHiS”, which enables the screening of interaction-defective alleles targeted for the entire length of a relatively long protein (more than 300 amino acids). We also employed the “Partitioned OPTHiS” method for the isolation of SRID mutant alleles over the whole region of HDAC5c (amino acids 680 to 1087). HDAC5c was partitioned into HDAC5cN (amino acids 680 to 831) and HDAC5cM (amino acids 829 to 1010), and then screened for SRID mutants through independent operation of OPTHiS (Fig 1A). In this case, each gap plasmid, G5N and G5M, harbored the HDAC5c region(s) that combined with the mutagenic PCR fragments (HDAC5cN and HDAC5cM) to generate the full-length HDAC5c domain in prey fusion proteins (Fig 1A).

### Isolation of SRID Mutants of HDAC4c and -5c Using “Partitioned OPTHiS”

**Fig 2:**
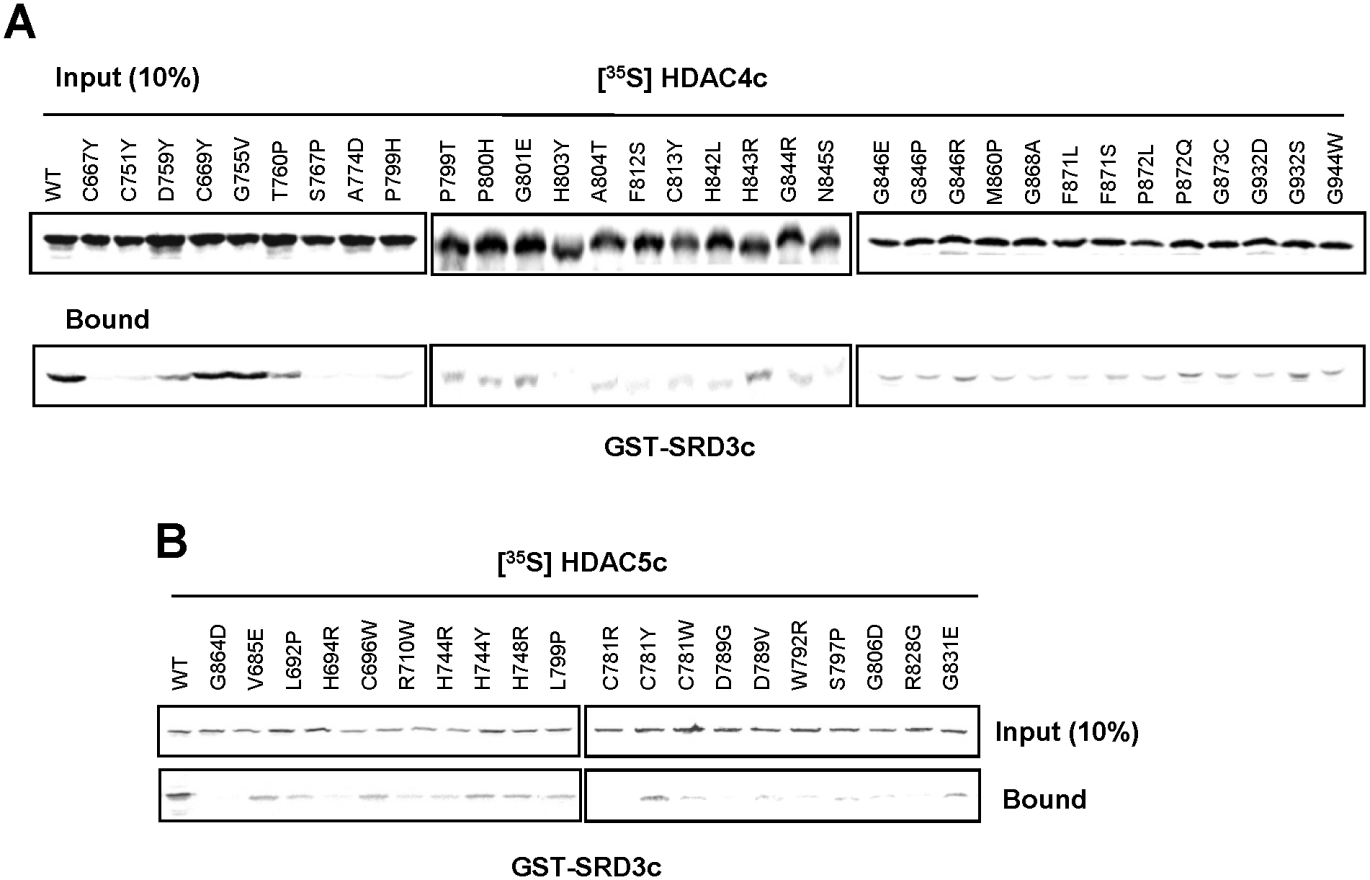
Defective interactions of SRD3c with the isolated HDAC4c and -5c mutants in GST pull-down assays. GST-SRD3c protein was purified and tested for interactions with the wild-type (WT) and mutant versions of ^35^S-labeled HDAC4c (A) or -5c (B) proteins. GST protein was used as the negative control. Input indicates 10% of the *in vitro* translated HDAC proteins used in the pull-down analysis.

As described in the materials and methods, we adopted a PCR-mediated random mutagenesis and gap-repair recombination method to generate mutant cell libraries for the HDAC4cN and HDAC4cT regions [45]. As candidates of non-interactor, a total of 29 and 23 white colonies were isolated on X-gal plates from the 2,700 and 2,200 transformants obtained, which contained missense mutations in the HDAC4cN and HDAC4cT regions, respectively (S1 Table). Through subsequent verification and sequencing analysis [45], a total of 36 SRID alleles for 29 residues of HDAC4c were finally isolated. Fig 1B shows the mutational sites and the amino acids changes in the isolated SRID mutants of HDAC4c (*upper panel*), from which it can be known that the mutation residues were distributed over the 300 amino-acid region of the N-terminal HDAC4c domain (spanning from Y658 to G944 position). Some mutants were isolated multiple times, and more than one amino acid change was observed at some residues, such as G846 (to Glu, Pro, Arg or Val). Next, the SRID phenotype of the HDAC4c mutants was confirmed in a quantitative yeast two-hybrid assay and *in vitro* GST-pull down analysis. After the pRS324UBG-HDAC4c mutants were transformed into the EGY48 strain bearing pRS325LexA-SRD3c, the binding strength between HDAC4c mutants and SRD3c was measured in a liquid β-galactosidase assay. A total of 33 mutants showed severe defects in SRD3c binding, whereas three mutants (C669Y, G755V, and G868A) displayed only a partial defect (S1A Fig). To confirm the SRID phenotype *in vitro*, [^35^S]-labeled HDAC4c mutant proteins were prepared using a TNT *in vitro* translation kit and then subjected to binding reaction with GST-fused SRD3c protein. Consistent with the yeast two-hybrid data, almost all of the mutants lost the ability to interact with SRD3c with the exception of C669Y and G755V mutants showing partial defects (Fig 2A).

HDAC5 is the closest homologue of HDAC4 among the class IIa HDACs, based on sequence similarity. To determine the general feature of the SRID alleles of class IIa HDACs, we next tried to isolate SRID mutants of HDAC5c. Based on the SRID allele information of HDAC4c, the 330 amino-acid region of the N-terminal of HDAC5c (amino acid 680 to 1084) was divided into two parts (HDAC5cN and HDAC5cM) and intensively screened for SRID mutants using “Partitioned OPTHiS,” as in the screening of HDAC4c mutants (Fig 1A). We independently isolated 27 and 42 white colonies as SRID mutants of HDAC5c from the 2,510 and 3,000 transformants harboring missense mutations in the HDAC5cN and HDAC5cM regions, respectively (S1 Table). After verification and sequencing of the mutant candidates, a total of 57 kinds of SRID alleles for 46 residues of HDAC5c were obtained (Fig 1B, *lower panel*). The mutational sites were distributed over the 320 amino-acids of the N-terminal region of the HDAC5c domain (spanning from G684 to G1005 position), similar to the results observed for the HDAC4c mutants. Among the 57 mutants, 20 representative mutants were selected, for which the SRID phenotypes were confirmed in the quantitative yeast two-hybrid and GST-pull down assays, as described (S1B Fig and Fig 2B). As expected, none of the mutants showed SRD3c-binding activity comparable to that of wild-type HDAC5c.

### Defective Interactions between SRD3c and SRID Mutants in HEK293 Cells

**Fig 3:**
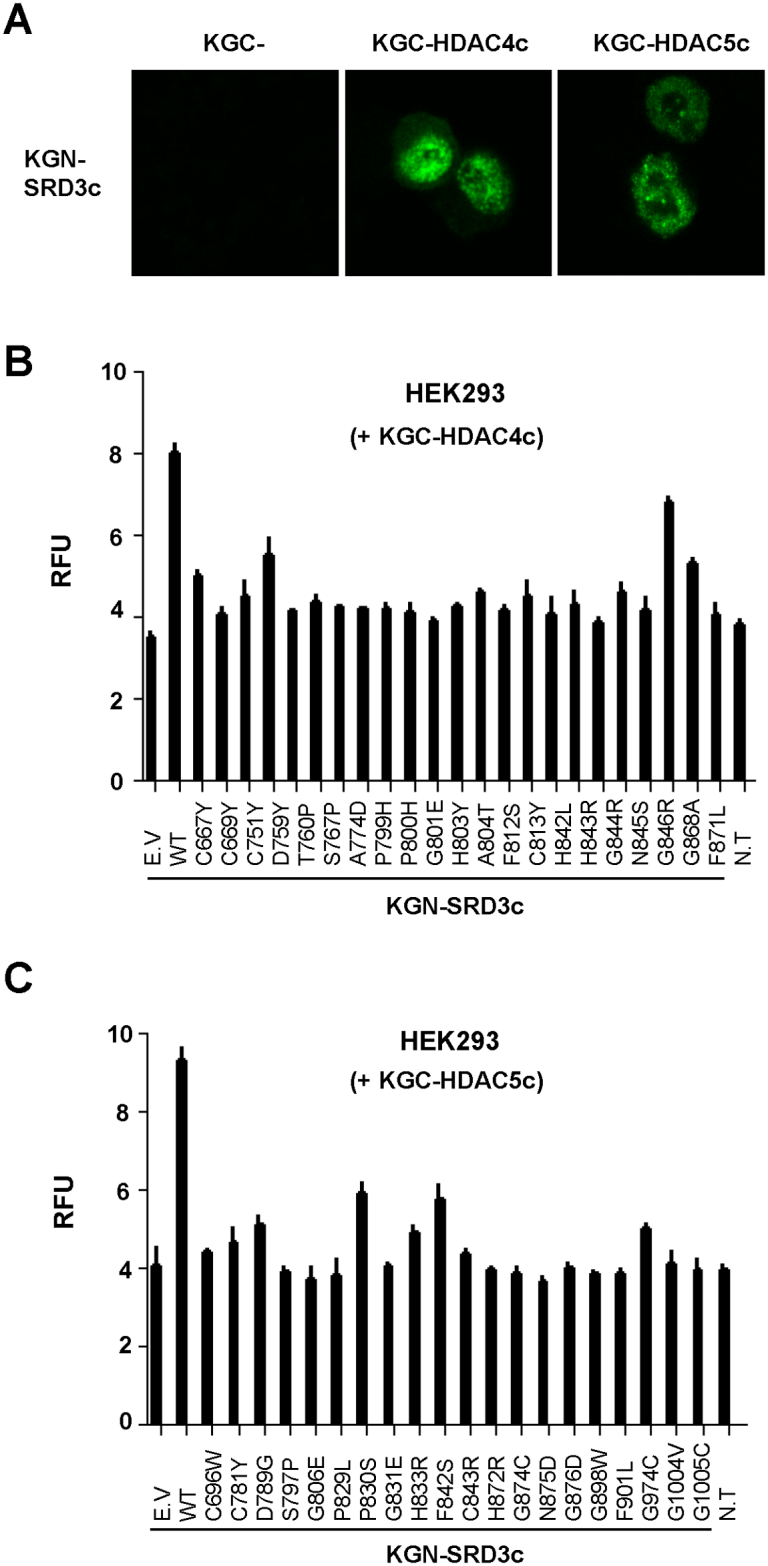
Defective interactions of SRD3c with SRID mutants of HDAC4c and -5c in HEK293 cells. **(A)** Confocal laser scanning microscope images illustrating the interactions between SRD3c and HDAC4c or -5c via BiFC assay. Fluorescent signals were mainly localized in the nucleus, and observed in the shape of speckles. *Magnification*: 180 X. **(B),(C)** Quantitative measurement of fluorescence signals in the BiFC assay, generated by the association of SRD3c with the indicated SRID mutants of HDAC4c (B) or -5c (C). The expression constructs for KGN-SRD3c (1 μg) and the indicated KGC-HDAC4c or -5c mutants (1 μg) were transiently cotransfected into HEK293 cells. After 48 hours of transfection, the fluorescent signals from whole cell lysates were measured with the use of a fluorescence spectrophotometer. E.V (empty vector) and N.T (no transfection) samples were used as negative controls. The *p*-values for all compared groups between wild-type and mutants are less than 0.01. RFU: relative fluorescence units.

To confirm the SRID phenotype of the isolated HDAC mutants in mammalian cells, the bimolecular fluorescence complementation (BiFC) assay was performed, which is a novel protein/protein interaction assay system that is independent of transcription mechanism. Expression plasmids for the N-terminal portion of the Kusabira Green protein (KGN) fused with SRD3c, as well as the C-terminal portion of the Kusabira Green protein (KGC) fused with HDAC mutants were constructed, followed by transient transfection of the plasmids into HEK293 cells. In this case, if the SRD3c and HDAC proteins associated with each other, green fluorescent signals would be generated from the reconstituted Kusabira Green protein. When KGN-SRD3c and the wild-type version of KGC-HDACs were expressed as the positive control of the BiFC assay, green fluorescent signals could be observed predominantly in the nucleus, with the shape of speckles via laser-scanning confocal microscopy (Fig 3A). This result is consistent with the previous observations that class IIa HDACs are colocalized with SMRT and HDAC3 in the nuclear matrix with a dot-like structure [36,46], confirming the physiological relevance of the *in vivo* interaction of SRD3c with HDAC4c or -5c (Fig 3A). Next, we measured the levels of fluorescence signals generated by interactions between SRD3c and the isolated SRID mutants via the BiFC assay using a fluorescence spectrophotometer (Fig 3B and 3C). The association of SRD3c with HDAC4c or -5c increased the fluorescent signal by about two-fold compared to the background signal generated by KGC empty vector (negative control). In contrast, with the exception of HDAC4c G846R mutant, which showed partial defect, none of the SRID mutants of HDAC4c and -5c were able to interact with SRD3c in the quantitative BiFC assay (Fig 3B and 3C), consistent with the yeast two-hybrid and GST pull-down data. Statistical analysis for all compared groups between wild-type and mutant HDACs revealed the significant differences among them (see *p*-values in S3 Table). Finally, the SRID phenotypes of some selected HDAC4c and -5c mutants were examined in a BiFC assay with confocal microscopy (S2 Fig). As expected, the signals from the nuclei of HEK293 cells transfected with the mutant versions of KGC-HDACs were significantly weakened or disappeared compared with those of the positive control. Overall, we successfully isolated SRID mutants of HDAC4c and -5c via partitioned operation of OPTHiS screening, targeted over the entire catalytic domains of these proteins.

### Positions and Features of SRID Mutations of HDAC4c and -5c

**Fig 4:**
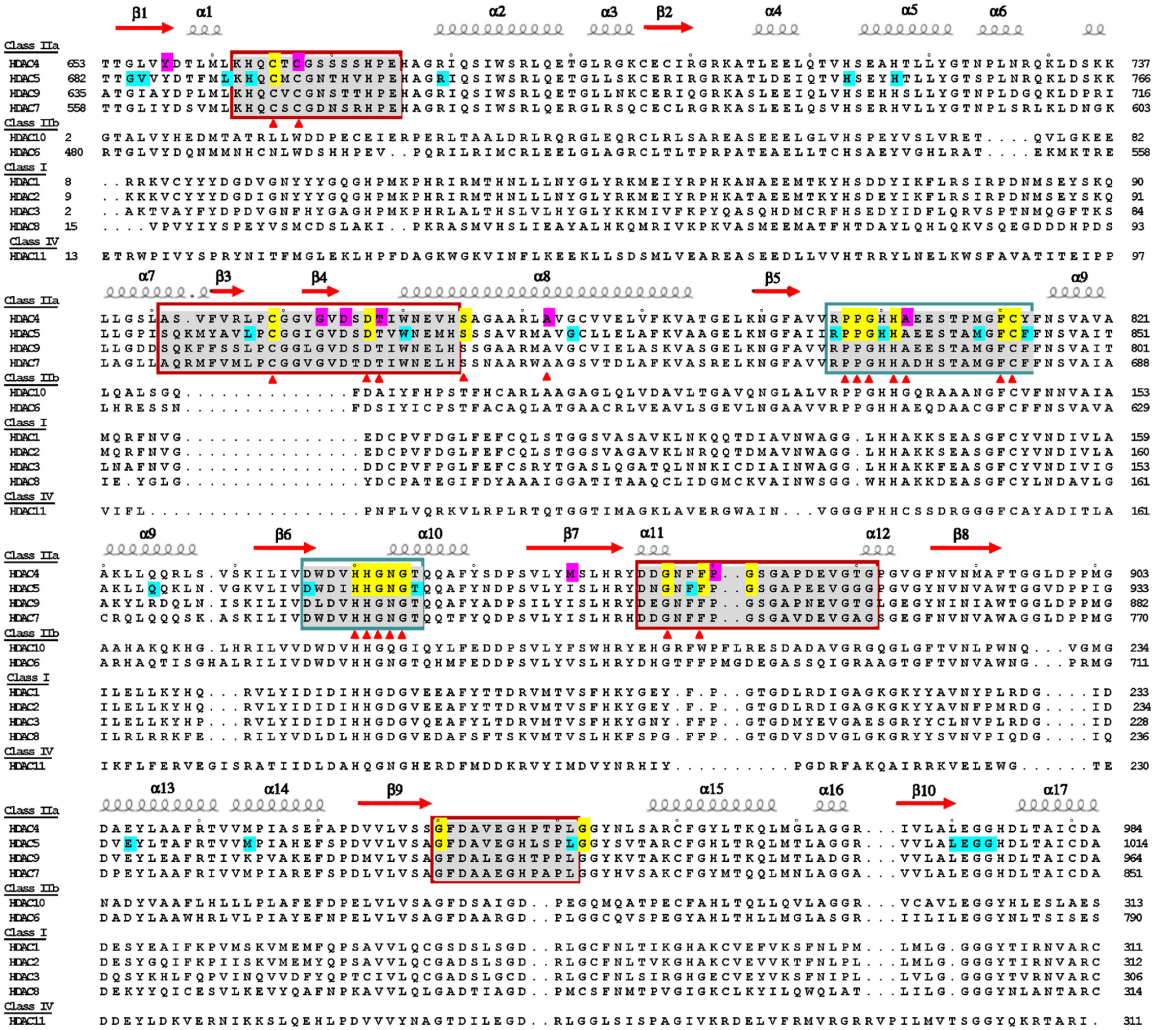
Positions of SRID mutations over the MSA of HDAC domains. Structure-based MSA of the catalytic domains from HDAC1 to HDAC11 was built using the ESPript 3.0 program, and secondary structural elements are shown for the inhibitor-bound HDAC4c structure (PDB code 2VQJ). The residues in pink and blue shades represent the SRID alleles specifically found in HDAC4c and -5c mutants, respectively. The residues with yellow shade indicate the SRID alleles commonly found at the same positions between HDAC4c and -5c sequences on the basis of MSA. *Red triangles*: the residues of HDAC4c mutants located on the surface region of the HDAC4c structure. *Blue Boxes* indicate hot-spot regions for SRID mutations which are commonly found in all classes of the HDAC family. *Red boxes* indicate hot-spot regions for SRID mutations which correspond to class IIa HDAC-specific regions.

The positions of the SRID alleles of HDAC4c and -5c over the structure-based multiple-sequence alignment (MSA) of HDAC domains from class I, -IIa, -IIb and -IV HDACs are presented in Fig 4. On the basis of MSA data, the positions of 20 SRID alleles were overlapped between 29 residues of HDAC4c mutations and 46 residues of HDAC5c mutations (gray boxes in Fig 1B and marked with yellow shade in Fig 4). These residues act as the general requirement for the interaction of class IIa HDACs with SRD3c, suggesting that the molecular determinants of HDAC4c interaction with SRD3c are similar to those of HDAC5c. Notably, some mutant residues were found exclusively in HDAC4c or -5c (purple shade for HDAC4c and blue shade for HDAC5c in Fig 4), suggesting these residues may act as the specific determinants required for the isotype-dependent interaction of class IIa HDACs with SRD3c. This interesting issue is left to be addressed in future work, and is currently under investigation.

As shown in Fig 4, most of the SRID mutants of HDAC4c and -5c were commonly found in the six regions of HDAC domains, labeled as the mutational hot-spots. Among them, two regions, corresponding to β5-α9 and β6-α10 loops, are conserved in all zinc-dependent HDAC enzymes (blue boxes), which accounted for nearly half of the SRID mutations. The rest of the SRID mutations were found as the mutational hot-spots in four regions of the HDAC domains (α1-α2 loop, β3-β4, α11-α12 loop and β9-α15 loop) which are specific to class IIa HDACs (red boxes), according to the structure-based MSA. These results indicate that the interaction of SRD3c with HDAC4c and -5c is largely mediated by the residues specific to class IIa HDACs, providing important clues to address the issue of how SRD3c specifically interacts with class IIa HDACs, but not with class I HDACs.

### The Rim Surface of the Catalytic Entry Site Is the Major SRD3c-Binding Surface of—HDAC4c and -5c

**Fig 5:**
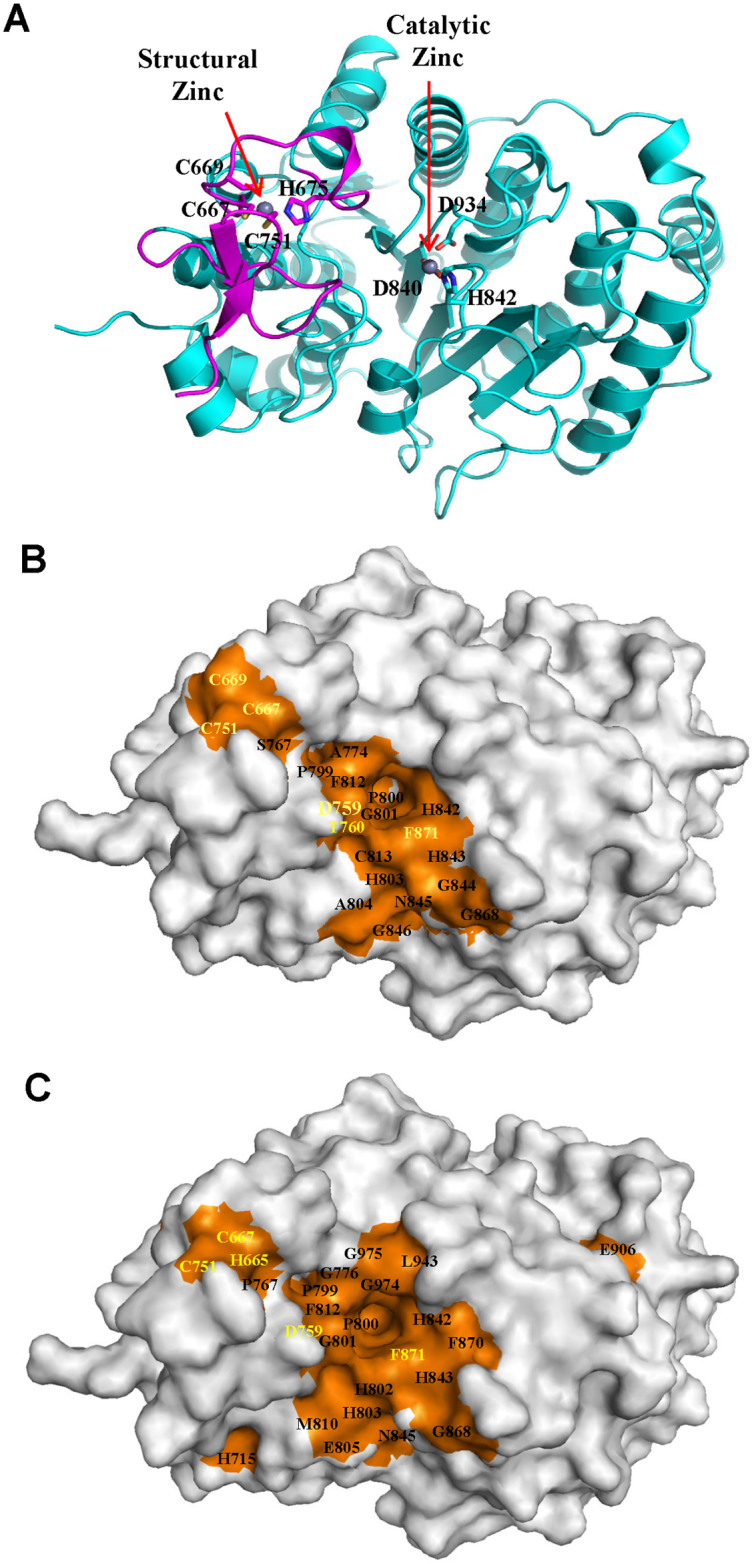
Surface presentation of SRID mutations on HDAC4c structure. **(A)** Ribbon diagram of HDAC4c structure (PDB code 2VQJ) [40]. The structural zinc-binding domain is shown by magenta. Two zinc ions and their chelating residues are drawn as spheres and sticks, respectively. **(B)** Positions of SRID mutations of HDAC4c are presented on the surface of the HDAC4c structure (PDB code 2VQJ). Among SRID mutants of HDAC4c, 21 residues located on the surface are indicated, and their surface positions are shown in orange. **(C)** Surface presentation of SRID mutations of HDAC5c at the corresponding positions of the HDAC4c structure. The positions of the SRID mutations of HDAC5c were changed to those of HDAC4c according to MSA data, and presented on the surface of HDAC4c structure in orange. The residues specific to class IIa HDACs are indicated in yellow color.

Next, we presented the SRID mutations on the surface of the HDAC4c structure because we reasoned that SRD3c associates with the surface region of the HDAC domains of class IIa enzymes. According to the crystal structure of inhibitor-bound HDAC4c (PDB code 2VQJ) [40], 21 residues (C667, C669, C751, D759, T760, S767, A774, P799, P800, G801, H803, A804, F812, C813, H842, H843, G844, N845, G846, G868 and F871) among the 29 identified residues of the HDAC4c mutations are located at the surface region of HDAC4c (bold letters in Fig 1B and red triangles in Fig 4). These residues were represented on the corresponding positions of the three-dimensional structure of HDAC4c, nicely generating two adjacent hot-spot regions at the surface of HDAC4c (orange in Fig 5B). Interestingly, one of the surface region, composed of four residues (C667 and C669 in α1-α2 loop, C751 in β3-β4 loop, and S767 in α8 region), was positioned at the structural zinc-binding domain known to exist only in class IIa enzymes (Fig 5A and 5B). In contrast, the remaining 17 residues generated a large surface region mainly composed of β5-α9 and β6-α10 loops on the rim of catalytic active site (Fig 5A and 5B). The SRID mutations of HDAC5c were also presented on the surface of the HDAC4c structure, as structural data for HDAC5c are not available. The positions of the SRID mutations of HDAC5c were changed to those of HDAC4c according to the MSA data, and displayed at the corresponding positions of the HDAC4c structure (orange in Fig 5C). In accordance with the HDAC4c data, one small region composed of four residues (H665/H694, C667/C696, C751/C781 and S767/S797 in HDAC4c/HDAC5c) appeared on the surface of the structural zinc-binding domain. Interestingly, 19 out of 25 surface residues of HDAC5c mutations were again mapped to the rim surface of the catalytic site, forming a major binding surface with a more extended region than that of HDAC4c (Fig 5B and 5C).

Taken together, our findings consistently indicated that most of the surface mutations were mapped to the rim of the catalytic entry site, rather than to the structural zinc-binding domain, which strongly suggests that this mutational hot-spot region is the major binding surface of SRD3c. This result is unexpected because the active site regions are evolutionally conserved among all HDAC members, making it unable to explain the reason why SRD3c specifically interacts only with class IIa HDACs. However, detailed analysis of the SRID mutant residues revealed that six residues (C667, C669, C751, D759, T760 and F871) of the HDAC4c surface mutations and five residues (H694, C696, C781, D789 and F901) of the HDAC5c surface mutations could be identified as class IIa HDAC-specific residues on the basis of the structure-based MSA (yellow letter in Fig 5). Among them, four residues (C696, C781, D789 and F901) of HDAC5c had overlapped positions with those of HDAC4c (C667, C751, D759 and F871). We suggest that these residues act as the specific requirements for SRD3c interaction with the class IIa HDACs, providing the molecular basis of their specific interactions.

### Functional Analyses of HDAC4c Mutants Containing SRID Allele

**Fig 6:**
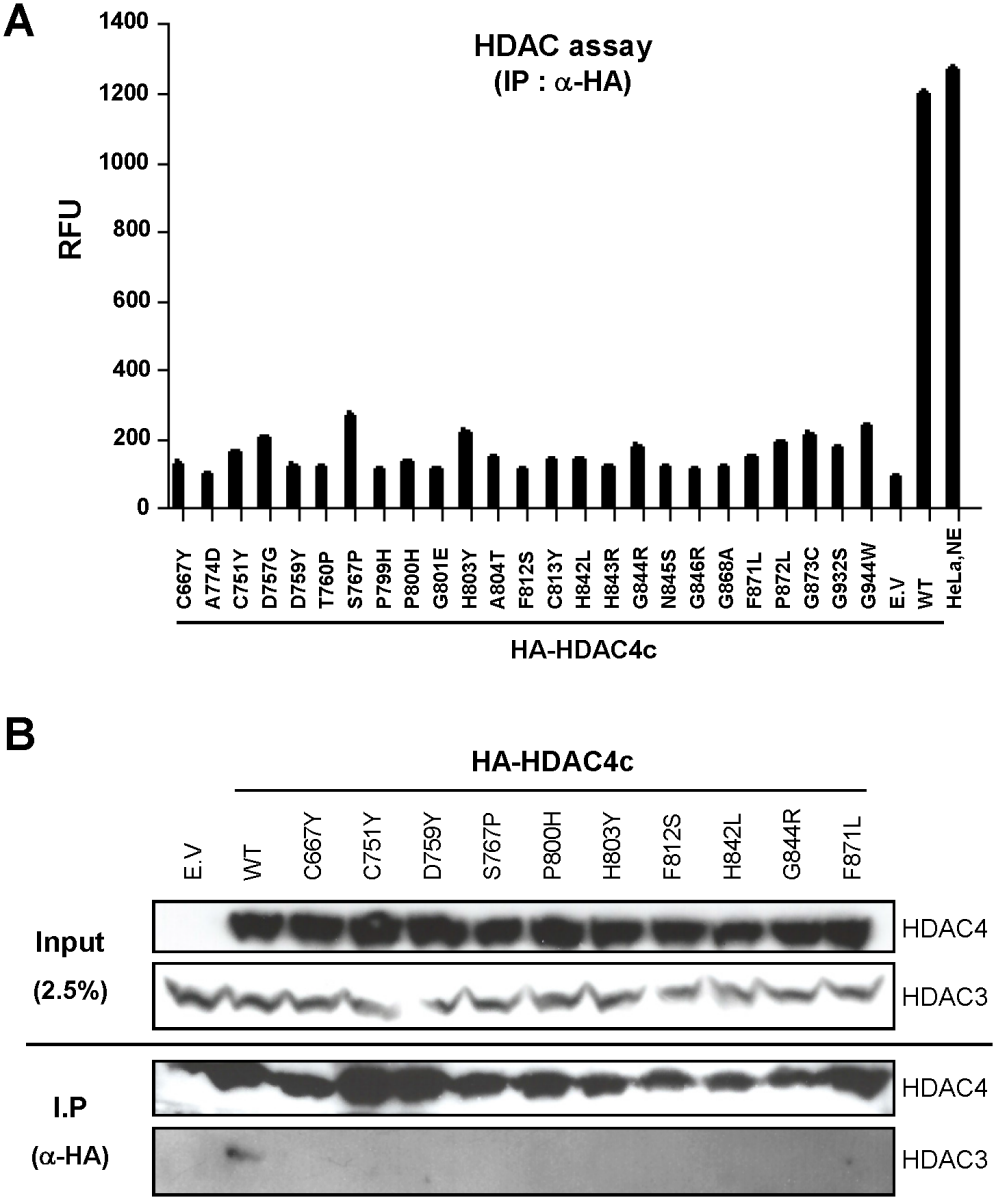
Loss of *in vitro* HDAC activity of immuno-purified HDAC4c mutants due to inability to associate with endogenous SMRT-HDAC3 complex. **(A)** *In vitro* HDAC assay and **(B)** co-immunoprecipitation assay for HDAC4c SRID mutant proteins immuno-purified from HEK293 cells. The HA-tagged versions of the indicated HDAC4c proteins were coexpressed with HDAC3 in HEK293 cells by transient transfection and purified from cell lysates (300 μg) with the use of agarose beads coupled with anti-HA-antibody. (A) The immune complex was subsequently subjected to *in vitro* HDAC assay using fluorescent-coupled Lys acetamide as a substrate. HeLa nuclear extract (1 μg) was used as the positive control. The *p*-values for all compared groups between wild-type and mutants are less than 0.0001. RFU: relative fluorescence units. (B) For co-immunoprecipitation assay, immunoprecipitates from 1 mg of whole cell lysate were prepared and analyzed for the presence of HA-HDAC4c and HDAC3 by immunoblot analysis.

As mentioned, the enzymatic activity associated with the endogenous class IIa HDAC complex is dependent on SMRT/NCoR-HDAC3, which is recruited through association of the HDAC domain [36]. To investigate the functional consequence of SRID mutations on HDAC4c, we next measured the *in vitro* HDAC activities of HDAC4 mutants purified from mammalian cells. To accomplish this, HA-tagged versions of 25 SRID mutants of HDAC4c were constructed and transiently expressed along with HDAC3 protein in HEK293 cells. The endogenous complex containing HDAC4c mutants was immuno-purified from the whole cell lysate using anti-HA antibody and then subjected to measurement of the *in vitro* HDAC activity toward Lys acetamide substrate, as described in the materials and methods. Intriguingly, the levels of HDAC activity of the immuno-purified HDAC4c mutants were dramatically decreased by eight to ten-fold compared to the wild-type HDAC4c (Fig 6A). The significant differences between the *in vitro* HDAC activities of wild-type and mutants HDAC4c were confirmed by statistical analysis (see *p*-values in S3 Table). We reasoned that the reduced HDAC activity of the immuno-purified HDAC4c mutants may be due to the compromised binding of the SMRT-HDAC3 complex to the HDAC4c mutants *in vivo*. To test this possibility, 10 surface mutants of HDAC4c harboring mutations at the class IIa HDAC-specific regions (C667, C751, D759, S767, and F871) or at the conserved regions among class I and IIa HDACs (P800, H803, F812, H842, and G844) were selected. After transient expression of HDAC3 and HDAC4c mutants in HEK293 cells, the endogenous complex containing wild-type or mutant HDAC4c was immuno-purified as described and subjected to immunoblot analysis to examine the presence of HDAC3 protein in the immune complex. Although the expression levels of HDAC4c mutants were comparable to that of the wild-type (Fig 6B, *upper panel*), HDAC3 displayed significantly compromised association with the HDAC4 mutants in the cell lysate of HEK293 cells (Fig 6B, *lower panel*), consistent with their low levels of *in vitro* HDAC activity (Fig 6A). From these observations, we concluded that the specific residues located at the rim surface of the catalytic entry site of HDAC4c are involved in the *in vivo* interactions with endogenous SMRT-HDAC3 complex.

**Fig 7:**
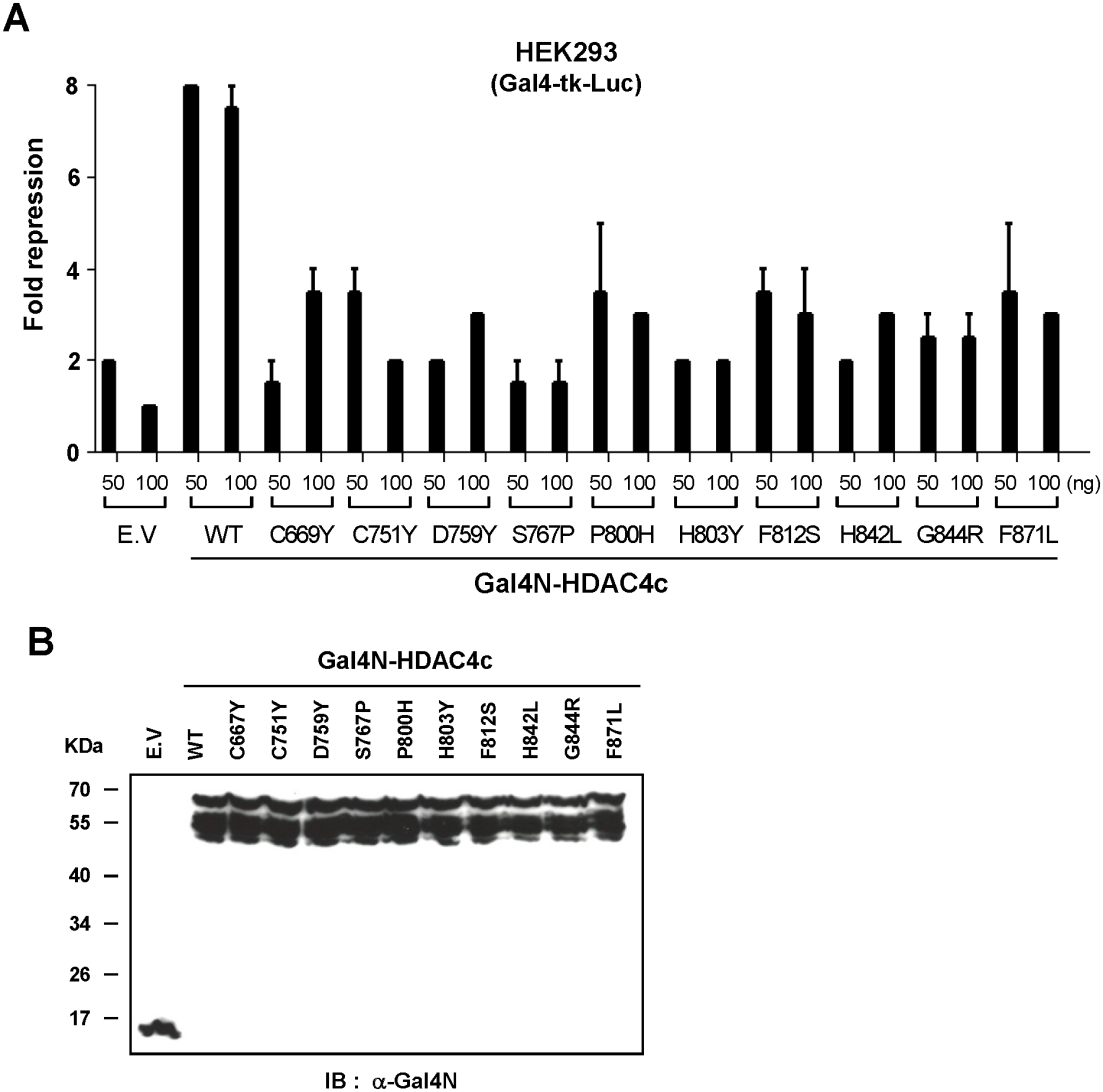
Transcriptional repressive activities of Gal4N-fused SRID mutants of HDAC4c. (A) Mammalian one-hybrid assay of HDAC4c mutants. HEK293 cells were cotransfected with the Gal4-tk-luciferase reporter plasmid (200 ng) and wild-type (WT) or the indicated mutants of Gal4N-HDAC4c plasmids (50 and 100 ng), together with the pCMV-β-gal vector (100 ng). After 48 hours of transfection, the luciferase activities were measured and normalized, as described in the materials and methods section. Fold repression indicates the mean ± S. E. value obtained from at least two independent experiments performed in duplicate. The *p*-values for all compared groups between wild-type and mutants are less than 0.05. RLU: relative luciferase activity. **(B)** The expression levels of wild-type (WT) or indicated mutants of Gal4N-HDAC4c proteins were examined by immunoblot analysis of whole cell lysates (15 μg) using anti-Gal4N mouse monoclonal antibody (Santa Cruz, sc-510; 1:3,000 dilution).

Finally, we investigated the functional consequences of SRID mutations on the transcriptional repressive activity of HDAC4c in a mammalian system. The Gal4N-fused version of wild-type or selected mutants of HDAC4c were prepared and cotransfected with the Gal4N-driven luciferase reporter plasmid into HEK293 cells. As shown in Fig 7A, the Gal4N-mediated recruitment of wild-type HDAC4c showed a maximum of 7-fold repression of reporter gene expression, suggesting the autonomous repressive activity of HDAC4c is provided by the endogenous SMRT-HDAC3 complex. In contrast, all HDAC4c mutants lost their autonomous repressive activities, displaying only 2 to 3-fold repression of luciferase expression (Fig 7A). Statistical analysis by student’s *t*-test revealed the significance of the relevant differences between the autonomous repressive activities of wild-type and mutants HDAC4c (see *p*-values in S3 Table). All of the Gal4N-fused HDAC4c proteins showed similar expression levels, as evidenced by immunoblotting using Gal4N antibody (Fig 7B). These results indicated that SRID mutants of HDAC4c lost their repressive function due to the crippled interactions with the endogenous SMRT-HDAC3 complex *in vivo*, in accordance with the previous results obtained by the co-immunoprecipitation assay (Fig 6B).

## Discussion

As mentioned in the introduction, the catalytic domain of class IIa HDACs exhibits a cryptic deacetylase activity toward acetylated lysine substrate, and plays a major role in the recruitment of the SMRT/NCoR-HDAC3 complex to repress the transcription of target genes *in vivo*. To gain an understanding of the molecular determinants of the specific interactions between class IIa HDACs and SRD3c, we isolated various kinds of SRID alleles for HDAC4c and -5c through the operation of “Partitioned OPTHiS,” targeting the entire HDAC domains.

### Partitioned OPTHiS for the Extensive Mutagenesis of the Entire Catalytic Domains of HDACs

In an effort to develop a method for the high-throughput analysis of protein interaction interfaces, we devised a modified yeast two-hybrid system, termed “OPTHiS,” which efficiently selects specific missense mutations that disrupt known protein-protein interactions [44]. To date, we have utilized this system for analysis of the interfaces of many protein-protein interactions between various nuclear receptors and their transcriptional coregulators, such as SMRT, NCoR, SRC-1, ASC-2 and TRAP220 [47–53]. In all cases, we tried to select missense mutant alleles of the coregulator proteins defective in nuclear receptor binding, which resulted in the identification of short motifs comprised of less than a dozen amino acids as the molecular elements of coregulators required for their specific binding to nuclear receptors. As mentioned above, our efforts to define the minimal region of the HDAC4c domain required for SRD3c binding revealed that the entire domain of HDAC4c (amino acids 651 to 1055) is necessary and sufficient for optimal binding to SRD3c. This result implicated that the intactness of the SRD3c-binding surface of HDAC4c requires the three-dimensional structure comprised of the whole catalytic domain, and a short motif within HDAC4c is not involved in SRD3c binding. In OPTHiS, the prey protein for mutant screening is expressed as a triple fusion between B42AD and GBD. Therefore, HDAC4c protein, which requires proper three-dimensional structure for SRD3c binding, may not be a suitable target for mutant screening by OPTHiS [45]. Fortunately, three-piece fusion forms of HDAC4c or -5c (B42-HDACs-GBD as a prey) can interact well with the LexA-fused SRD3c protein (bait) in a yeast two-hybrid system, enabling the screening of SRID mutants of these HDACs using OPTHiS. However, there is a restriction on the length of the prey protein, owing to the difficulty of maintaining the optimal mutation rate by PCR-mediated random mutagenesis with increasing prey size. Thus far, we have successfully analyzed prey proteins of up to 200 amino acids. We considered that the catalytic domain of class IIa HDACs (about 400 amino acids) is too long to allow isolation of the SRID mutants via the conventional OPTHiS screening method. Therefore, we solved this problem by developing a novel strategy, named “Partitioned OPTHiS”, in which a relatively long prey protein (more than 300 amino acids) is divided into fragments of less than 200 amino acids, and the interaction-defective alleles for each fragment are screened by the independent operation of OPTHiS. In Partitioned OPTHiS, the design of the gap plasmid is critical, because *in vivo* gap repair by the homologous recombination between the terminal regions of the PCR fragment (mutagenic target) and the linearized gap plasmid should produce the full-length prey protein, which can interact with the bait protein. For example, the gap plasmid G4N employed herein was designed to harbor the HDAC4cT region between the B42AD and GBD parts of the pRS324UBG plasmid (Fig 1A). During *in vivo* gap repair, the mutagenic HDAC4cN fragment was inserted between the B42AD and HDAC4cT regions of the linearized G4N plasmid, resulting in the generation of the full-length HDAC4c domain harboring the random mutation(s) at the HDAC4cN region. Using the “Partitioned OPTHiS” method, we were able to successfully isolate various kinds of SRID alleles of HDAC4c and -5c via extensive mutagenesis of their entire catalytic domains. We propose that the “Partitioned OPTHiS” method can be generally applied for the characterization of protein-binding interfaces, in which the three-dimensional domain structure composed of more than 300 amino acids is involved, via the rapid and efficient isolation of interaction-defective missense mutants.

### Interaction Surface of Class IIa HDACs with SMRT Corepressor

According to the MSA data, most of the SRID mutants of HDAC4c and -5c were commonly found in six regions of the HDAC domains as mutational hot-spots (Fig 4). Two of the regions are conserved in all HDAC members (β5-α9 and β6-α10 loops), while the remaining four are specific to class IIa HDACs (α1-α2 loop, β3-β4 region, α11-α12 loop and β9-α15 loop). A previous report showed that some catalytic core mutants of HDAC4 exhibited compromised binding to the SMRT-HDAC3 complex *in vivo*, and consequential loss of *in vitro* HDAC activity when immuno-purified from HEK293 cells [36]. Among these mutants, three mutations located in the β5-α9 loop (H803Y, H842L and N845S) were isolated in our SRID mutant screening, verifying that these residues, positioned in the evolutionally conserved catalytic pocket, are involved in SRD3c binding.

The surface display of SRID mutant residues on the corresponding positions of the HDAC4c structure allowed identification of two adjacent hot-spot regions as the SRD3c-binding surface of class IIa HDACs: one minor surface located on the structural zinc-binding domain, and one major region corresponding to the rim surface of the catalytic pocket of HDAC enzymes (Fig 5). As noted, the structural zinc-binding domain exists exclusively in class IIa members, and is considered to participate in substrate recognition and modulation of enzyme activity via protein interaction(s) with the regulatory proteins [39–41]. In addition, a previous report indicated that mutation of the residues (e.g. C669A) coordinating the structural zinc ion prevented its association with the endogenous SMRT-HDAC3 complex, pointing to a key role of the structural zinc-binding domain in the recruitment of binding partner proteins [40]. All of these results strongly suggested the structural zinc-binding domain as a good candidate for the specific interactions of class IIa HDACs with SRD3c. This notion left open the possibility that both of the two adjacent surface regions mapped by our SRID mutant screening are directly involved in SRD3c binding via simultaneous interactions with two independent parts of the SRD3c region.

However, our analysis of the SRID alleles consistently indicated that most of the surface mutations (17 out of 21 residues of HDAC4c and 19 out of 25 residues of HDAC5c) were mapped to the rim surface of the catalytic pocket, rather than to the structural zinc-binding domain (Fig 5). This observation raises another intriguing possibility that SRD3c solely interacts with the major binding surface on the catalytic pocket, but not with the minor surface region on the structural zinc-binding domain. According to this view, the SRID phenotype caused by mutations at the structural zinc-binding domains can be explained by “indirect effects," in which the conformational changes of the structural zinc-binding domain could affect the structural stability of the neighboring catalytic pocket region and indirectly lead to the loss of SRD3c-binding ability observed in these mutants. The previous structural analyses support this notion, that the two adjacent regions have an intimate structural relationship and that the flexibility of the structural zinc-binding domain might be involved in this connection. For example, the binding of some active site inhibitors and/or substrates to the catalytic pocket of HDAC4c may affect the conformation of its structural zinc-binding domain [40]. This opinion is based on the observation that the apo-form of the active site mutant of HDAC4c (H976Y) showed considerable differences in the conformation of the structural zinc-binding domain (“closed” conformation) compared with the inhibitor-bound structures (“open” conformation). This structure resembles the structure of the substrate-bound HDAC8 complex, regarded as the active conformation of the HDAC domain [40]. We propose that the conformational change of the structural zinc-binding domain may affect the intactness and SRD3c-binding capacity of the genuine interacting surface formed on the catalytic pocket region. Conversely, it is also possible that the structural zinc-binding domain is the unique and genuine binding partner of SRD3c, and that the mutational effects of the major surface mutants are indirect. We considered this case as unlikely for the following two reasons. Firstly, there was a widespread distribution of major surface mutations from the catalytic entry site to the bottom wall region of the catalytic pocket (β5-α9 loop and α11- α12 region), which is spatially distant from the structural zinc-binding domain (Figs 4 and 5). Secondly, there was a striking difference between the numbers of mutations in the two SRD3c-binding surfaces, in which most of the residues of the surface mutations (17 out of 21 residues of HDAC4c and 19 out of 25 residues of HDAC5c) formed the large SRD3c-binding surface on the catalytic pocket region. Taken together, we suggest the major mutational hot-spot region formed on the active site pocket to be the SRD3c-binding surface of class IIa HDACs, though we cannot rule out the possibility of direct involvement of the structural zinc-binding domain in this interaction.

### Molecular Determinants of HDAC4c and -5c Required for Their Specific Binding to SRD3c

Among the surface residues of HDACs required for SRD3c binding, three residues (C667, C669, and C751 in HDAC4c) located on the structural zinc-binding domain were identified as class IIa HDAC-specific residues on the basis of the structure-based MSA. In the major binding surface on the catalytic pocket, three residues (D759, T760, F871) of HDAC4c and two of HDAC5c (D789 and F901) were proven to be specific to class IIa HDACs. Considering the binding mode and the structural basis typically observed in many protein-protein interactions, it is unlikely that all of the surface residues required for SRD3c binding are directly involved in this interaction. Rather, we suppose that a few residues, including class IIa HDAC-specific ones (e.g. D759, T760 or F871 of HDAC4c), actually participate in SRD3c-binding, while the rest of the surface residues indirectly affect the intactness of the SRD3c-binding surface. Determination of the molecular requirements of SRD3c for HDAC binding and detailed analysis of the binding interface by a crystallographic study will eventually clarify the binding mode of SRD3c to class IIa HDACs and the structural basis of the specific interactions.

Class IIa HDACs have been implicated as potential therapeutic targets for many human diseases, including cancers, metabolic disorders, pathological cardiac hypertrophy, acute ischemic injury, inflammatory or autoimmune conditions and central nervous system disorders [11,35,54–60]. Therefore, the development of small molecule activators or inhibitors selective for class IIa HDACs would have a great impact on the treatment of these diseases, with minimization of off-target effects. To date, most HDAC inhibitors share the common structural characteristics of a pharmacophore model comprised of a metal binding moiety, linker region and surface recognition domain [61]. Thus, all of these inhibitors can bind simultaneously to the rim, channel and active site of the catalytic pocket, thereby inhibiting HDAC activity by blocking substrate access. Most inhibitors effective for class IIa HDACs also have the same pharmacophore structure, with direct binding to this catalytic site. The active site region bound by these inhibitors is evolutionally conserved among the all zinc-dependent HDAC enzymes [61], explaining why these inhibitors do not show isotype or class-specific effect. Using a mutagenesis approach, we identified the surface region of HDAC4c responsible for the interaction with SRD3c, which acts as the binding module specific to class IIa HDACs. Our results strongly suggest that the surface region of the catalytic pocket of class IIa HDACs, rather than active site channel, could be a more plausible and reasonable target for the development of class IIa HDAC-specific inhibitors, because this region has structural features distinguishable from those of class I enzymes.

Recently, novel inhibitors selective for class IIa HDACs were developed, and their therapeutic effects on Huntington’s disease were evaluated [62]. Interestingly, these HDAC inhibitors were designed to exploit the lower pocket of the catalytic entry site that shows a characteristic of the class IIa HDACs, but not found in other HDAC classes. From the co-crystal structure of HDAC4c bound by these inhibitors, we found that the targeted lower pocket region is overlapped with the major SRD3c-binding surface mapped by our mutagenesis study. Furthermore, the inhibitors dock on the lower pocket region via key interactions with the HDAC4 residues, including D759, H803, F812, D840, H842, F871 and D943 [62]. Most of these residues (D759, H803, F812, H842 and F871) were found in the major SRD3c-binding surface revealed herein, raising the intriguing possibility that the surface region composed of these residues directly participates in the binding of SRD3c by means of structural characteristics specific to class IIa HDACs.

In conclusion, our mutagenesis study provided the molecular basis for the specific interactions of SRD3c with HDAC4 and -5, as well as structural insights into the binding interface, which may be helpful for the design of modulators specific to class IIa HDACs.

## Supporting Information

S1 Fig Defective interactions of SRD3 with the isolated HDAC4c and -5c mutants in yeast-two hybrid assay. The expression constructs for LexA-fused SRD3c and B42AD-GBD-fused HDAC4c (A) or -5c (B) mutants were co-transformed into EGY48 strains containing a *Lac*Z reporter plasmid, pSH18-34. Transformants were subjected to liquid β-galactosidase assay to measure the binding strength between SRD3c and HDAC mutants. β-galactosidase activity was shown as the representative of three independent experiments. E.V (empty vector) samples were used as the negative control. The *p*-values for all compared groups between wild-type and mutants are less than 0.001
(TIF)

S2 Fig Confocal analysis of the images produced by the interactions between SRD3c and SRID mutants of HDAC4c in BiFC assay. BiFC assay was performed to assess the interactions between SRD3c and the indicated SRID mutants of HDAC4c (A) and -5c (B) in HEK293 cells. The expression constructs for KGN-SRD3c (500 ng) and KGC-HDAC4c or -5c mutants (500 ng) were transiently cotransfected into HEK293 cells. After 48 hours of transfection, the cells were fixed on micro cover-slides and the green fluorescence signals from the cells were observed using a laser-scanning confocal microscope. *Magnification*: 60 X.
(TIF)

S1 Table Transformation and screening of SRID mutants by OPTHiS. Each of the mutagenic PCR products of the HDAC domains was generated and co-transformed with the indicated gap plasmid into strain YOK400 carrying the pSH18-34 reporter as well as the bait plasmid, pRS325LexA-SRD3c. His^**+**^ transformants were obtained after a 3-day incubation at 30°C on glucose media lacking histidine. Transformants were picked onto plate media containing X-gal but lacking histidine, and the yeast colonies showing white color were isolated as candidates of non-interactor.
(DOCX)

S2 Table List and sequences of oligonucleotides used in this study. (DOCX)

S3 Table The *p*-values obtained by student’s *t*-test for the compared groups in graphs. (DOC)
