# Supplementary figures and images for: Mutagenesis Study Reveals the Rim of Catalytic Entry Site of HDAC4 and -5 as the Major Binding Surface of SMRT Corepressor
Source: PLoS One. 2015 Jul 10;10(7):e0132680. doi: 10.1371/journal.pone.0132680 (PMC4498904; doi:10.1371/journal.pone.0132680)

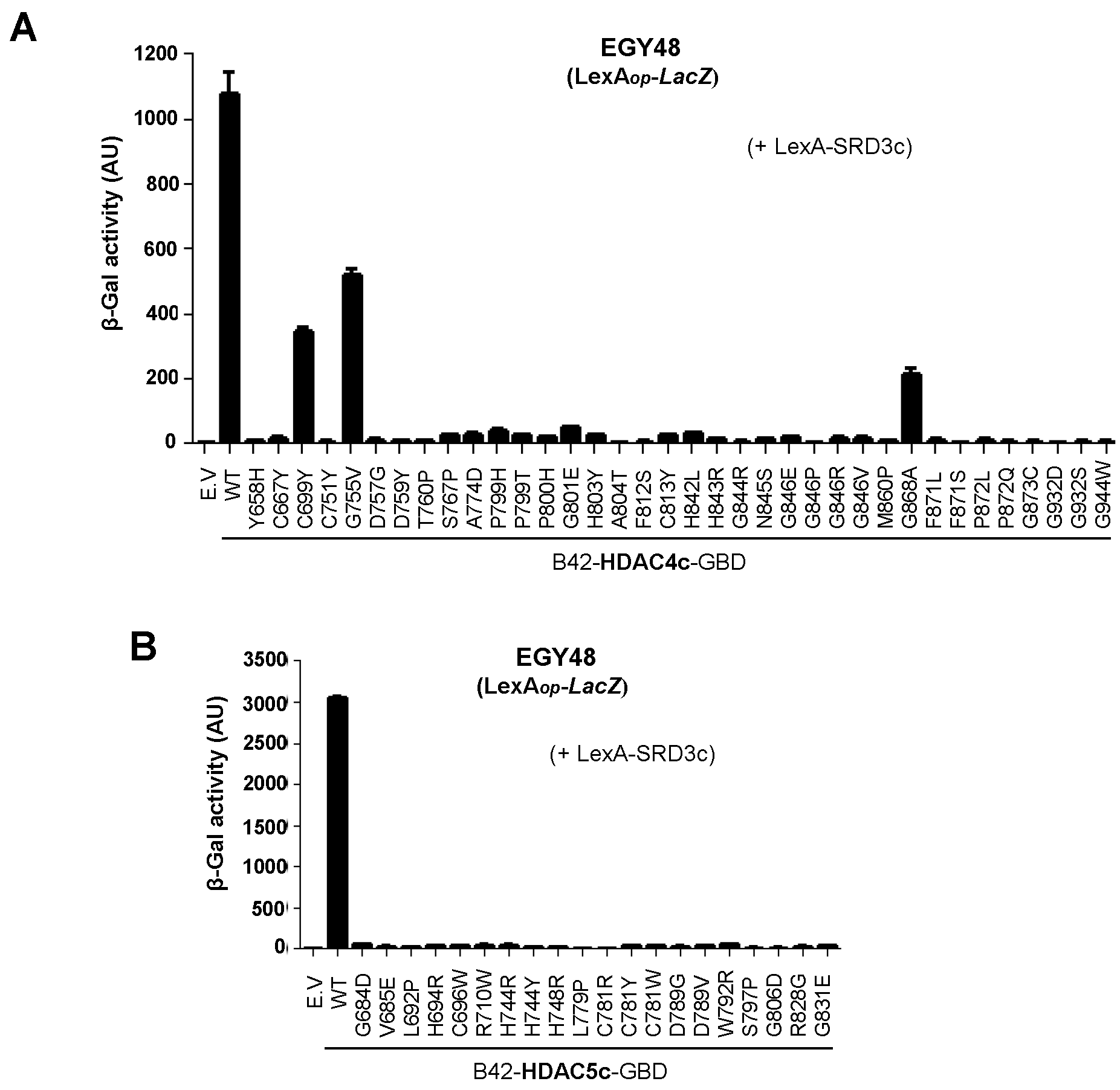

Supplement: S1 Fig — The expression constructs for LexA-fused SRD3c and B42AD-GBD-fused HDAC4c (A) or -5c (B) mutants were co-transformed into EGY48 strains containing a LacZ reporter plasmid, pSH18-34. Transformants were subjected to liquid β-galactosidase assay to measure the binding strength between SRD3c and HDAC mutants. β-galactosidase activity was shown as the representative of three independent experiments. E.V (empty vector) samples were used as the negative control. The p-values for all compared groups between wild-type and mutants are less than 0.001 (TIF) [file pone.0132680.s001.tif]

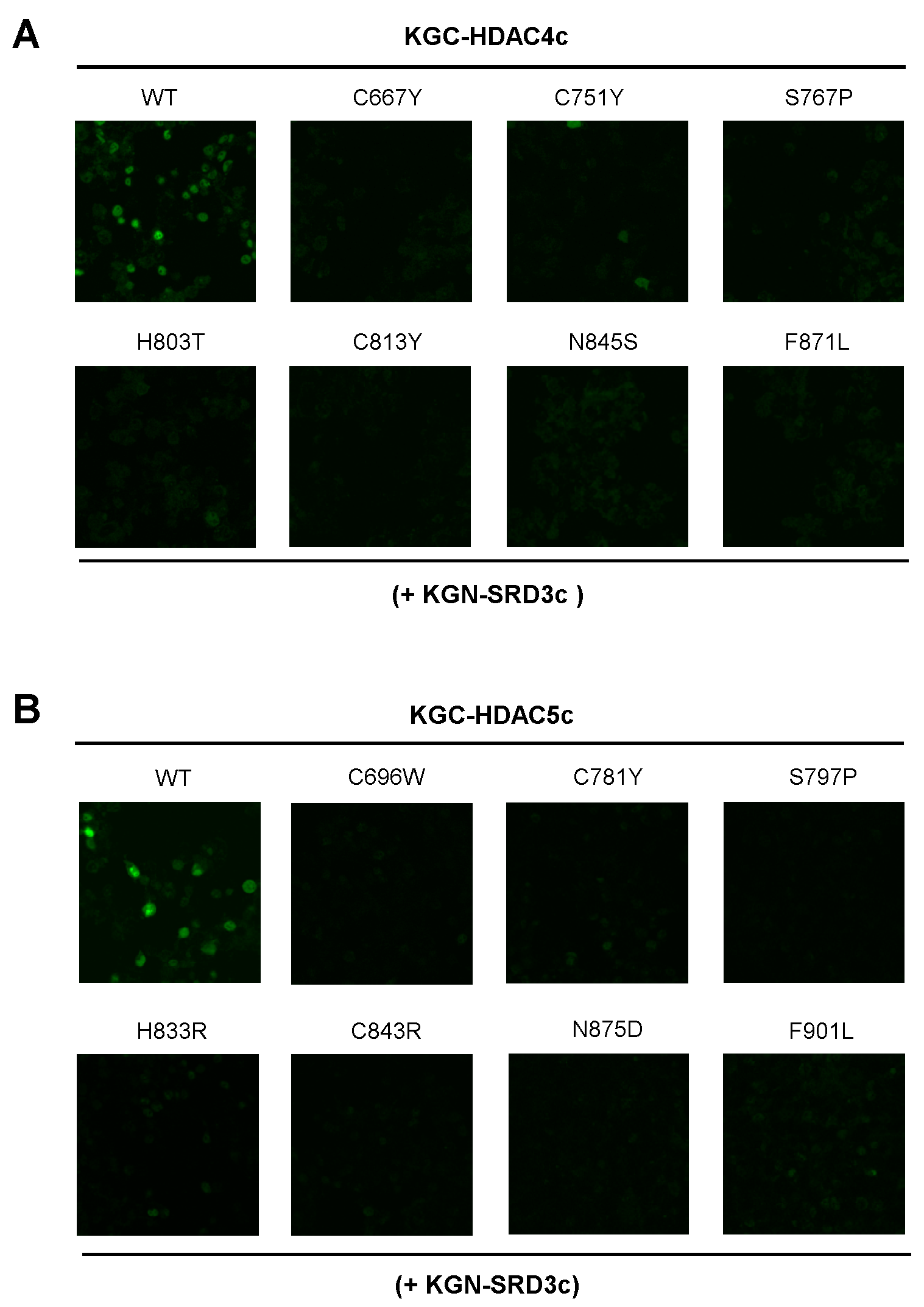

Supplement: S2 Fig — BiFC assay was performed to assess the interactions between SRD3c and the indicated SRID mutants of HDAC4c (A) and -5c (B) in HEK293 cells. The expression constructs for KGN-SRD3c (500 ng) and KGC-HDAC4c or -5c mutants (500 ng) were transiently cotransfected into HEK293 cells. After 48 hours of transfection, the cells were fixed on micro cover-slides and the green fluorescence signals from the cells were observed using a laser-scanning confocal microscope. Magnification: 60 X. (TIF) [file pone.0132680.s002.tif]
