# Supplementary material for: Mutagenesis Study Reveals the Rim of Catalytic Entry Site of HDAC4 and -5 as the Major Binding Surface of SMRT Corepressor
Source: PLoS One. 2015 Jul 10;10(7):e0132680. doi: 10.1371/journal.pone.0132680 (PMC4498904; doi:10.1371/journal.pone.0132680)
Supplement: S1 Table — Each of the mutagenic PCR products of the HDAC domains was generated and co-transformed with the indicated gap plasmid into strain YOK400 carrying the pSH18-34 reporter as well as the bait plasmid, pRS325LexA-SRD3c. His+ transformants were obtained after a 3-day incubation at 30°C on glucose media lacking histidine. Transformants were picked onto plate media containing X-gal but lacking histidine, and the yeast colonies showing white color were isolated as candidates of non-interactor. (DOCX) [file pone.0132680.s003.docx]

Supplementary Table 1. Transformation and screening of SRID mutants by OPTHiS.

|  |  | | | |  | |
| --- | --- | --- | --- | --- | --- | --- |
| Transforming DNAs  Gap plasmid PCR products | Total no. of  transformants | Mutation rate (%)  (no. of white/no. picked) |  |  | |  |
|  |  |  |  |  | |  |
| G4N HDAC4cN | 5.4 x 10^3^ | 1.07 (29/2700) |  |  | |  |
| G4T HDAC4cT | 4.1 x 10^3^ | 1.04 (23/2200) |  |  | |  |
| G5N HDAC5cN | 5.7 x 10^3^ | 1.08 (27/2510) |  |  | |  |
| G5M HDAC4cM | 6.0 x 10^3^ | 1.40 (42/3000) |  |  | |  |
