# Supplementary material for: Mutagenesis Study Reveals the Rim of Catalytic Entry Site of HDAC4 and -5 as the Major Binding Surface of SMRT Corepressor
Source: PLoS One. 2015 Jul 10;10(7):e0132680. doi: 10.1371/journal.pone.0132680 (PMC4498904; doi:10.1371/journal.pone.0132680)
Supplement: S2 Table — (DOCX) [file pone.0132680.s004.docx]

Supplementary Table 2. List and sequences of oligonucleotides used in this study

| Name | DNA sequence (5’ to 3’) |
| --- | --- |
| RD3cF | GGGAGATCTTAGAGGGTGGCATGTCTGTG |
| RD3cR | GGGCCATGGTCAGTAGCAGGCACGTTCCAG |
| 4cF | GGGGAATTCAGGTTCACGACAGGCCTC |
| 4cR | GGGGAGATCTACCGTCTCGGCTTCTTCGTT |
| 4cTF | GAATCCATGGATCCGACGATGGGAAC |
| 4cN3R | CCAGATCTACTAGTGTAGCGGTGGAGGGAC |
| 4cR2 | GGGTCTAGATCACGTCTCGGCTTCTTCGTT |
| 4cNR2 | GGGAGATCTGCGTCTCCCATGGGGGGGTCCAG |
| 4cT2F | TGAGCAAAGATCCTCATCGT |
| 5c2F | GGGGAATTCCTCTTCACCACAGGTGTG |
| 5cT2R | GGGGGATCCTTGCTCACAGTCTCGGCCTC |
| 5cT2F | GGGGAATTCTTCGAGATCTGTGCGCATGGCAGTG |
| 5c2NR | GTCGACGGGGCCGGATGATGGCAAATCC |
| 5c2CF | GTCGACAGCCCTGGAGGGAGGCCATGAC |
| 5c3R | TGTGGATTCCTCGGCGTGGTG |
| 5c4F | GAGATGCACTCCTCCAGTGCT |
| 5c4R | CTGCAAGACTGCCTCATCCAA |
| B42F | CCAGCCTCTTGCTGAGTGGAG |
| GBDIR | CGGTTTTTCTTTGGAGCAC |
