# Supplementary material for: Mutagenesis Study Reveals the Rim of Catalytic Entry Site of HDAC4 and -5 as the Major Binding Surface of SMRT Corepressor
Source: PLoS One. 2015 Jul 10;10(7):e0132680. doi: 10.1371/journal.pone.0132680 (PMC4498904; doi:10.1371/journal.pone.0132680)
Supplement: S3 Table — (DOC) [file pone.0132680.s005.doc]

**Supplementary Table 3. The *p-*values obtained by student’s *t*-test for the compared groups in graphs.**

**Fig. 3B (WT vs. mutants)**

| **C667Y** | **C669Y** | **C751Y** | **D759T** | **D759Y** | **T760P** | **S767P** | **A774D** | **P799H** | **P800H** | **G801E** |
| --- | --- | --- | --- | --- | --- | --- | --- | --- | --- | --- |
| 0.0015 | <0.0001 | 0.0048 | 0.0041 | 0.0002 | 0.0016 | 0.0003 | 0.0003 | 0.0003 | 0.0017 | 0.0001 |
| **H803Y** | **A804T** | **F812S** | **C813Y** | **H842L** | **H843R** | **G844R** | **N845S** | **G846R** | **G868A** | **F871L** |
| <0.0001 | 0.0007 | 0.001 | 0.0015 | 0.0017 | 0.0037 | 0.0009 | 0.0022 | 0.001 | 0.0058 | 0.0004 |

**Fig. 3C (WT vs. mutants)**

| **C696W** | **C781Y** | **D789G** | **S797P** | **G806E** | **P829L** | **P830S** | **G831E** | **H833R** | **F842S** |
| --- | --- | --- | --- | --- | --- | --- | --- | --- | --- |
| 0.0017 | 0.0078 | 0.0015 | 0.0011 | 0.0052 | 0.008 | 0.0027 | 0.0016 | 0.0017 | 0.0003 |
| **C843R** | **H872R** | **G874C** | **N875D** | **G876D** | **G898W** | **F901L** | **G974C** | **G1004V** | **G1005C** |
| 0.003 | 0.0012 | 0.0037 | 0.0011 | 0.0024 | 0.0023 | 0.0026 | 0.0018 | 0.0053 | 0.0043 |

Fig. 6A (WT vs. mutants)

| **C667Y** | **A774D** | **C751Y** | **D757G** | **D759Y** | **T760P** | **S767P** |
| --- | --- | --- | --- | --- | --- | --- |
| <0.0001 | <0.0001 | <0.0001 | <0.0001 | <0.0001 | <0.0001 | <0.0001 |
| **P799H** | **P800H** | **G801E** | **H803Y** | **A804T** | **F812S** | **C813Y** |
| <0.0001 | <0.0001 | <0.0001 | <0.0001 | <0.0001 | <0.0001 | <0.0001 |
| **H842L** | **H843R** | **G844R** | **N845S** | **G846R** | **G868A** | **F871L** |
| <0.0001 | <0.0001 | <0.0001 | <0.0001 | <0.0001 | <0.0001 | <0.0001 |
| **P872L** | **G873C** | **G932S** | **G944W** |  | | |
| <0.0001 | <0.0001 | <0.0001 | <0.0001 |  | | |

Fig. 7A (WT vs. mutants)

|  | **C669Y** | **C751Y** | **D759Y** | **S767P** | **P800H** | **H803Y** | **F812S** | **H842L** | **G844R** | **F871L** |
| --- | --- | --- | --- | --- | --- | --- | --- | --- | --- | --- |
| *50 ng* | 0.0024 | 0.0049 | 0.0005 | 0.0022 | 0.0346 | 0.0005 | 0.0045 | 0.0005 | 0.0029 | 0.0354 |
| *100 ng* | <0.0001 | 0.0027 | 0.0041 | <0.0001 | 0.0041 | 0.0027 | 0.0348 | 0.0041 | 0.0005 | 0.0041 |
